# Supplementary material for: Epigenetic CpG duplex marks probed by an evolved DNA reader via a well-tempered conformational plasticity
Source: Nucleic Acids Res. 2023 Mar 15;51(12):6495–506. doi: 10.1093/nar/gkad134 (PMC10325892; doi:10.1093/nar/gkad134)
Supplement: gkad134_Supplemental_Files [file gkad134_supplemental_files.zip › SI_clean2.pdf]

Supporting Information

Epigenetic CpG Duplex Marks Probed by an Evolved DNA Reader via a Well-Tempered Conformational Plasticity

Himanshu Singh[1], Chandan K. Das[2], Benjamin C. Buchmuller[1], Lars V. Schäfer[2]*, Daniel Summerer[1]*, Rasmus Linser[1]*

[1] Department of Chemistry and Chemical Biology, TU Dortmund University, Otto-Hahn-Str. 4a, 44227 Dortmund, Germany.

[2] Theoretical Chemistry, Ruhr University Bochum, Universitätsstr. 150, 44801 Bochum, Germany.

* To whom correspondence should be addressed.

Rasmus Linser – Department of Chemistry and Chemical Biology, TU Dortmund University, 44227 Dortmund, Germany; https://orcid.org/0000-0001-8983-2935; Tel: +49 (0) 231 755 3910; Email: rasmus.linser@tu-dortmund.de

Daniel Summerer – Department of Chemistry and Chemical Biology, TU Dortmund University, 44227 Dortmund, Germany; https://orcid.org/0000-0002-3019-7241; Email: daniel.summerer@tu-dortmund.de

Lars V. Schäfer – Theoretical Chemistry, Ruhr University Bochum, Universitätsstr. 150, 44801 Bochum, Germany; https://orcid.org/ 0000-0002-8498-3061; Email: lars.schaefer@ruhr-uni-bochum.de

Present Address: Himanshu Singh, Department of Chemical Sciences, Indian Institute of Science Education and Research (IISER) Berhampur, Berhampur, Ganjam, Odisha, 760010, India.
Email: himanshus@iiserbpr.ac.in

Table of Contents:

Extended Materials and Methods 3

Electrophoretic mobility shift assays (EMSA) and determination of apparent binding affinity (KD).... 3

Overexpression and purification of MBD variants 3

Protein expression for NMR spectroscopy 3

NMR sample preparation and measurements 4

^{15}N Relaxation experiments 4

^{15}N Relaxation dispersion 5

^{15}N CEST experiments 6

NMR Structure Calculation 6

Table S1A: Details of the MBD variants used in this study 8

Table S1B: Details of the oligodeoxynucleotide probes used in this study 8

Fig. S1: Electrophoretic mobility shift assay (EMSA) 10

Table S2: Model estimates from EMSA gel shift images 10

Fig. S2: 2D [^{15}N–^{1}H]–HSQC of the wild-type and triple MBD mutant 12

Fig. S3: Overlaid ^{15}N relaxation data of the wild-type and triple mutant TAN 13

Fig. S4: Chemical shift changes as a function of temperature difference. 14

Fig. S5: Correlation between experimental and back-calculated backbone amide RDCs 14

Table S3: NMR structural statistics for the ensembles of 10 refined conformers of TAN 15

Fig. S6: Selected ^{15}N relaxation dispersion profiles of the wt MBD 16

Fig. S7: Selected ^{15}N relaxation dispersion profiles of the triple MBD mutant (TAN). 16

Fig. S8: k_{ex} as a function of residue for the wt and triple MBD mutant (TAN). 17

Table S4: Model selection, kinetic and thermodynamic exchange parameters from ^{15}N CPMG 17

Fig S9: Representative ^{15}N CEST profiles of the indicated amide groups of TAN 19

Fig. S10: Arrhenius plot 20

Fig. S11: Representative ^{15}N CEST profiles 20

Fig. S12: Structure and dynamics characterization of the double mutant MBD (TVN). 21

Fig. S13: Incident of partial unfolding in MD simulations 22

Fig. S14: Assigned 2D ^{15}N-^{1}H HSQC of hmC/mC DNA-bound TAN mutant. 22

Fig. S15: Selected chemical-shift changes in apo TAN versus TAN:hmC/mC DNA 23

Fig. S16: Overlays of apo TAN and complex seen by either NMR or MD 23

Fig. S17: Visualization of the specific interactions of the hmC/mC reader TAN with its target DNA 24

Fig. S18: Differential backbone plasticity of wt, TVN, and TAN mutants 24
Fig. S19: H-bond stability between Arg and guanosine in the complex... 25
Fig. S20: Identification of the R111-D121 salt-bridge in the wt and TAN mutant MBD.................... 25
Fig. S21: Salt bridges formed in the TAN, TVN, and wt 26
References: 26

Extended Materials and Methods

Electrophoretic mobility shift assays (EMSA) and determination of apparent binding affinity (KD):

MBD–DNA binding was assessed as described previously (1). The presence of mC and hmC were confirmed by mass spectrometry. In brief, gel shift images were quantified with ImageQuant TL v8.1 1D Gel Analysis (GE Healthcare) and the band intensity data analyzed with R v4.01. Using the Levenberg-Marquardt nonlinear least-squares algorithm, the following binding model was fitted:

$$[RL] / [L]_0 = b + B \cdot ([R]_0 + [L]_0 + K_D - (([R]_0 + [L]_0 + K_D)^2 - 4[R]_0[L]_0)^{1/2}) / 2 / [L]_0 \quad (1)$$

where $[L]_0$ is the total ligand concentration (2 nM) and $[R]_0$ the concentration of MBD in each lane, b the background estimate and B a correction factor for variation in active probe constraint to $0.55 < B < 1.1$; $[RL] / [L]_0$ is the fraction of bound duplex per lane. Fitted curves are shown in supplementary figure S1D and respective model estimates in Table S2.

Overexpression and purification of MBD variants*:*

The MBD proteins were recombinantly expressed in BL21-Gold(DE3) (Agilent Technologies) and purified as described earlier (1). For protein:DNA complexes, the purified proteins were added in a ratio of 1:1 with the respective DNA constructs (see Table S1B), synthesized by solid-phase DNA synthesis as described earlier (1). Epigenetic modifications were incorporated as the respective phosphoramidites. A correct modification was verified by mass spectrometry.

Protein expression for NMR spectroscopy:

Starting from a 2 L expression culture (25 °C, 240 rpm, overnight) in M9 medium set up according to a protocol modified from Marley et al. (2) supplemented with ^{13}C glucose (Cambridge Isotope Laboratories, Andover, MA, U.S.) and ^{15}N-ammonium chloride, the bacterial pellet was resuspended in 30 mL extraction buffer and treated with lysozyme in presence of PMSF for 60 min on a wheel-shaker at 4 °C. The suspension was extracted by pulse sonication or on a high-shear microfluidizer homogenizer. Insoluble debris was removed at 30,000 × *g* for 45 min at 4 °C. The cleared supernatant was sterile-filtered (0.4 µm syringe filter) and loaded on a 10 mL column self-packed with HisPurTM Ni-NTA resin (Thermo Fisher) connected to an ÄKTA FPLC (Fast Protein Liquid Chromatograph, GE Healthcare, Solingen, Germany). The mixture was separated at 1 mL/min flow rate of binding buffer containing 5 – 90 mM imidazole (0 – 100%) in 80 min. Fractions containing the MBP–MBD fusion protein were combined for dialysis. For NMR analyses, the solubility tag was cleaved by

adding His-free TEV protease to the combined eluates of the column purification after a first dialysis (3.5 kDa MWCO) against binding buffer without imidazole (20 mL eluate against 2 L buffer). The dialysis buffer was exchanged once. Then, the His-free TEV and the solubility tag were removed over the same Ni-NTA column as before using a gradient of 0–30% over 150 min. The MBDs were eluted with 100% 90 mM imidazole in binding buffer and again the desired fractions combined. The combined fractions were concentrated over an Amicon® centrifugal filter device (3.5 kDa MWCO; Merck) to 1 – 2 mL for size-exclusion chromatography and loaded onto a HiPrep 26/60 Sephacryl® S-200HR dextran (Merck) column. The MBD domain was polished at a flow rate of 1 mL/min of the final buffer; the desired fractions combined and concentrated as before.

NMR sample preparation and measurements:
For NMR studies, uniformly $^{13}C/^{15}N$-doubly-labeled protein was produced in minimal (M9) media supplemented with $^{15}NH_4Cl$ and ^{13}C-glucose as the sole source of nitrogen and carbon, respectively, and purified as described above. Purified uniformly-$^{13}C/^{15}N$ wild-type (KVS) and its double (TVN) and triple (TAN) mutants were prepared in a mixed solvent of 90% H_2O and 10% ^2H_2O (50 mM sodium phosphate, 50 mM NaCl, pH 6). All NMR experiments were carried out with protein concentrations of ~0.5 mM on a Bruker Avance 800 MHz NMR spectrometer equipped with a 5 mm cryogenically cooled triple-resonance probe and a pulse-field gradient. A suite of 3D double- and triple-resonance NMR experiments were performed for sequence-specific ^1H, ^{13}C and ^{15}N backbone resonance assignments as discussed earlier (3, 4). The ^1H chemical shifts were referenced to the external standard 2, 2-dimethyl-2-silapentene-5-sulfonates (DSS), while ^{15}N and ^{13}C chemical shifts were calibrated indirectly. The near-complete ^1H, ^{13}C and ^{15}N resonance assignments of MBD mutant MeCP2 protein has been deposited to the BMRB (http://www.bmrb.wisc.edu) under the accession number 51020. A similar sequence for the wild-type protein can be found under accession code 4280. The chemical-shift perturbations were measured as $[(\Delta\delta_H)^2+(\Delta\delta_N/10)^2]^{1/2}$, where $\Delta\delta_H$ and $\Delta\delta_N$ signify the changes in $^1H^N$ and ^{15}N chemical shifts, respectively. The factor 10 for ^{15}N chemical shift was taken as the normalization factor since the broad range of nitrogen chemical shifts is approximately ten times that of proton chemical shifts for the backbone amides in folded proteins. In addition to the backbone experiments, we recorded 3D experiments such as HCCH-TOCSY (mixing time = 12 ms), [^{15}N, ^1H]-NOESY-HSQC (mixing time = 100 ms), aliphatic and aromatic [^{13}C, ^1H]-NOESY-HSQC (mixing time = 120 ms) for almost complete assignment of ^1H, ^{13}C and ^{15}N side chain resonances, dihedral angle restraints, and NOE derived distance constraints for the 3D structure calculation. Water refined structural ensembles of the mutants were calculated from the experimental distance restraints using ARIA software (5). $^1H/^{15}N$-RDCs were collected using Pf1 filamentous phage (purchased from Asla Biotech) with a concentration of 11 mg/mL as an alignment medium, resulting in $^1H^N$-$^{15}N^H$ residual dipolar couplings between -15 and +15 Hz. The RDCs were determined via IPAP HSQC experiments (6). Alignments of the sample was confirmed by a D_2O quadrupole splitting of 8 Hz. PALES (7) was used to calculate the alignment tensor, resulting in a tensor magnitude of 7.18 Hz and a rhombicity of 0.087.

^{15}N Relaxation experiments:
The backbone ^{15}N T_1 relaxation measurements at 800 MHz were acquired at 291 K using recovery delays of 50, 100, 200, 300, 500, 700, 900 and 1100 ms. The ^{15}N T_2 measurements were carried out

with the same acquisition parameters using a CPMG pulse sequence (8) with relaxation delays of 5, 20, 35, 50, 70 and 90 ms. Steady-state [^{15}N, ^{1}H] heteronuclear-NOE measurements were carried out with and without proton saturation during the relaxation delay. In these NOE-experiments, 5 s of relaxation delay and 3 s of proton saturation (or 8 s of relaxation delay only) were used. The heteronuclear-NOE values were determined as the ratio of the peak intensities measured from the spectra acquired with and without proton saturation. NMR spectra were processed using TopSpin4.0.8 (Bruker BioSpin) and analyzed using CARA (9) and CCPN (10).

^{15}N Relaxation dispersion:

Constant-time ^{15}N-CPMG (CT-CPMG) relaxation dispersion experiments (11) were measured at 291 K at 800 MHz Larmor frequency. In addition, in the case of TAN, constant-time ^{15}N-CPMG data were recorded at two fields, 800 and 700 MHz, also at 283 K. Experiments were performed with a constant-time delay of 60 ms, and 9 variable CPMG frequencies (ν_{CPMG}) ranging from 50 to 2000 Hz were collected. Besides, for each data set the frequencies 750 and 50 Hz were repeated for estimation of errors in $R_{2,\mathrm{eff}}$, and a reference spectrum without constant time delay (T_{CPMG} = 0) was recorded. For each 2D dataset corresponding to one CPMG frequency, 128 and 2048 complex points in the indirect and direct dimensions, respectively, were collected with 32 scans. 2D datasets for all frequencies were measured in a scan-interleaved fashion. The recycle delay of 1.5 s was used, giving rise to a net acquisition time of approximately 2 h per data set.

NMR relaxation data were processed using Topspin 4.0.8. Peak intensities were quantified and visualized using SPARKY (12). The effective amide ^{15}N transverse relaxation rate at each CPMG frequency was calculated according to the relation of effective transverse relaxation rate ($R_{2,\,eff}$),

$$R_{2,eff} = \frac{-\ln\left(\frac{I}{I_0}\right)}{T} \qquad (2)$$

where I is the peak intensity, I_0 is the corresponding intensity in a reference spectrum recorded without the CT-CPMG relaxation period and T is a constant time delay. Data were analyzed individually using the NESSY software package (13) to obtain the kinetic parameters of interest, corresponding to a two-site exchange process. NESSY fits the profiles to no-exchange and fast-exchange mathematical models. It then chooses the best model for each residue based on the corrected Akaike information criterion.

Model 1: no exchange

$$R_{2,\,eff} = R_2^{0} \qquad (3)$$

Model 2: two states, fast exchange

$$R_{2,\,eff} = R_2^{0} + \phi_{ex}/k_{ex}[1.0 - (4\ \nu_{CPMG}/k_{ex})*\tanh(k_{ex}/(4\ \nu_{CPMG}))] \qquad (4)$$

Model 3: two states, slow exchange (Richard-Carver equation)

$$R_{2,\,eff} = R_2^{0} + k_{ex}/2 - \nu_{CPMG}\cosh^{-1}[D_{+}\cosh(\eta_{+}) - D_{-}\cosh(\eta_{-})] \qquad (5)$$

Fitting of individual models to experimental effective transverse relaxation rates ($R_{2,eff}$) is performed using the Levenberg-Marquardt algorithm via the sum of least squares by minimizing the χ^2 target function as follows:

$$\chi^2 = \sum_i \frac{(R^{expt,i}_{2,eff} - R^{calc,i}_{2,eff})^2}{(\sigma^{R^{expt,i}_{2,eff}})^2} \qquad (6)$$

For global analysis, a two-state model (i.e. with the same kinetic parameters for all dispersion profiles considered) was fitted to the data using the program SHEREKHAN (14), which minimizes the target function by numerically propagating mathematical equations such as the Carver-Richards, Luz-Meiboom, and Bloch-McConnell models. Global analysis for the wt combined the following residues K107, Q110, R111, F157, V159 and T160. On the other hand, for TAN we combined the following residues for global exchange parameters: G103, T105, T109, Q110, R111, K112, S113, G114, S116, A117, G118, Y120, D121, Y123, I125, G129, K130, A131, F132, R133, N134, E137, L138, A140, Y141, F142, V145, G146, T148, S149, and N153.

^{15}N CEST experiments:

^{15}N CEST experiments were performed with a 0.5 mM ^{15}N labeled sample. The pulse sequence was implemented as described previously (15). For triple-mutant MBD construct TAN, two sets of CEST experiments were performed at nominal ^{15}N B_1 fields of 15 Hz and 30 Hz. The B_1 field was scanned across 64 points of the ^{15}N spectral width, from 134 to 99 ppm.

The ^{15}N carrier was centered on a peak that was well separated in the ^{1}H dimension. A 2D experiment was performed where the length of the ^{15}N B_1 field was varied during t_1. For both, the 30 and 15 Hz fields, 128 complex points were acquired in t_1. The data was processed with Topspin4.0.2. Peak heights were extracted by fitting the series of spectra with the SPARKY. CEST profiles were fit with the software Dynamics Center (version 2.7.2) to extract the exchange parameters. CEST data were fitted to a two-state exchange between ground (G) and excited (E) states, G ⇌ E, based on the Bloch-McConnell equation (16).

NMR Structure Calculation:

The 3D solution structure of MBD triple mutant and complex were determined using the following NMR constraints: (i) Dihedral angle constraints derived using TALOS-N (17) with the knowledge of individual ^{1}H^N, ^{15}N, ^{13}Cα, ^{13}Cβ, ^{13}CO chemical shift values as inputs. A total of 108 and 114 ϕ and ψ dihedral angle constraints were used for the apo TAN and TAN:hmC/mC complex, respectively. (ii) Cross peaks in NOESY spectra were identified and automatically assigned using ARIA 2.3 (5). The upper-bound distance constraints were set to 6.0 Å. Residual dipolar couplings were obtained via ^{1}H/^{15}N-RDCs using Pf1 filamentous phage (purchased from Asla Biotech) with a concentration of 10 mg/mL as an alignment medium, resulting in ^{1}H-^{15}N RDCs between -15 and +15 Hz. ^{1}H/^{15}N-RDCs were collected using via IPAP HSQC experiments (6), with an alignment tensor calculated via the PALES (7) software. Alignments of the sample was confirmed by a D_2O quadrupole splitting of 8 Hz. PALES was used to calculate the alignment tensor, resulting in a tensor magnitude of -7.18 Hz and a rhombicity of 0.087. Structural statistics were summarized in Sable S1. With all these restraints as input, the 3D structures were calculated using the simulated annealing

protocol in ARIA 2.3. A total of 100 structures were calculated, from which 10 structures with lowest target function and no distance or angle violations were selected. These 10 conformers with lowest target function were further refined in explicit water with 25 $^{1}H/^{15}N$-RDC restraints for the apo protein, NMR-derived distance restraints and angle restraints using the inbuilt ARIA 2.3 CNS program. The program PSVS-1.4 (http://www.psvs-1_4.nesg.org) was used to validate the quality of the selected ensemble of lowest-energy structures of apo TAN and complex. The 3D coordinates of individual atoms thus obtained were deposited in the PDB (pdb IDs: 8AJR and 8ALQ, see Table S3). The structure figures were prepared using Pymol (The PyMOL Molecular Graphics System, Version 1.8 Schrödinger, LLC) (18) and UCSF Chimera.

Molecular Dynamics Simulations:

We built our simulated system of wt MBD with a methylated DNA (namely, wt:mC/mC) from the previously reported X-ray coordinates of DNA:MBD cocrystal structure (PDB ID 3C2I) (19). Similarly, the systems of the double mutant (TVN) and the triple mutant TAN with an asymmetric hmC/mC DNA (namely, TVN:hmC/mC and TAN:hmC/mC, respectively) were constructed from the same X-ray structure by replacing side chains of mutated residues that includes K109T and S134N for TVN and, K109T, V122A, and S134N for TAN. The nucleobase of 5-hydroxymethylated cytosine (hmC) was built by replacing a proton of methyl group of mC by a hydroxy (OH) group. On the other hand, the starting structures of apo proteins (namely apo WT, TVN, and TAN) were obtained by simply removing DNA coordinates from the previous DNA:MBD constructs. All aforementioned structural modifications were performed using Chimera software (20).

The all-atom ff99bsc0 (21, 22) Amber force field was employed for describing proteins and nucleic acids including mC and hmC. Additional force field parameters corresponding to methyl and hydroxymethyl modifications in cytosine nucleobase were adopted from the general Amber force field (GAFF), while the RESP partial charges obtained by fitting to the electrostatic potential computed at the B3LYP/6-31G* level were used for mC and hmC. Each DNA-bound system was placed into a box with a size of ca. $8.5 \times 7.0 \times 9.2$ nm^3 with ca. 13600 TIP3P water molecules, whereas each apo protein system was kept in a box with a size of ca. $7.0 \times 6.0 \times 7.2$ nm^3 with ca. 7500 TIP3P water molecules. The net charge of each system was neutralized by adding appropriate numbers of Na^{+} and Cl^{-} ions. Periodic boundary conditions (PBC) were applied in all three dimensions.

All MD simulations were carried out using the Gromacs simulation package (23), version 2019.2. Each system was energy minimized at the force field level and then proceeded to the MD simulations. A simulated annealing of 200ps (the system was heated from 0 K to 300 K) was carried out at constant volume and constant temperature (NVT ensemble), followed by 500 ps equilibration at constant pressure and constant temperature (NPT ensemble) with harmonic position restraints (with force constants of 1000 kJ/mol/nm^2) applied to all heavy atoms of protein (and DNA for protein:DNA complexes). This was followed by a 500 ps equilibration at NPT ensemble conditions with harmonic position restraints (with force constants of 1000 kJ/mol/nm^2) applied to all backbone atoms of protein (and DNA for protein:DNA constructs). Subsequent to the restraint equilibration phase, the restraint potentials were switched off and the production runs (in the NPT ensemble) were carried out in where all atoms were allowed to move. A total number of five MD simulations for each system were

initiated using different random seeds (from a Maxwell Boltzmann distribution at 300 K) for generating the initial velocity. Each MD trajectory was 500 ns long, thus yielding a total simulation time of 2.5 μs for each system.

The simulations were performed using the leap-frog algorithm with time steps of 2 fs. During NPT simulation, the temperature of 300K was kept constant by coupling the system to a velocity rescaling thermostat with a time constant of 0.1 ps, while a constant pressure of 1 bar was maintained using the Berendsen barostat (24) with time constant of 2 ps. The nonbonded Coulomb and Lennard-Jones 6,12 interactions were described with a buffered Verlet pair list (25) with potentials smoothly shifted to zero at a cutoff of 1.0 nm, while the long-range electrostatics were treated with the particle-mesh-Ewald (PME) method (26). The LINCS (27) and the SETTLE (28) constraint algorithms were used to constrain all bonds involving H atoms and the internal degrees of freedom of the water molecules, respectively.

Table S1A: Details of the MBD variants used in this study:

Name	Protein	Sequence
wt	p1388	MeCP2
TAN	p1859	MeCP2[K109T/V122A/S134N]-His6
TIN	p2687	MeCP2[K109T/V122I/S134N]-His6
TLN	p2688	MeCP2[K109T/V122L/S134N]-His6
TGN	p2689	MeCP2[K109T/V122G/S134N]-His6
TVN	p2706	MeCP2[K109T/S134N]-His6

Table S1B: Details of the Oligodeoxynucleotide probes used in this study:

Name	Assay	Type	Sequence (5'-to-3')	Modified bases
mC/mC	EMSA	Duplex	TTTTTTTTTTTXGTTTTTTTTTTT<br>[6-FAM]AAAAAAAAAAAYGAAAAAAAAAAA	X=5mC,<br>Y=5mC
hmC/mC	EMSA	Duplex	TTTTTTTTTTTXGTTTTTTTTTTT<br>[6-FAM]AAAAAAAAAAAYGAAAAAAAAAAA	X=5mC,<br>Y=5hmC
hmC/mC	NMR	Hairpin	GATGAXGTAAAGTTTTCTTTAYGTCATC	X=5mC,<br>Y=5hmC

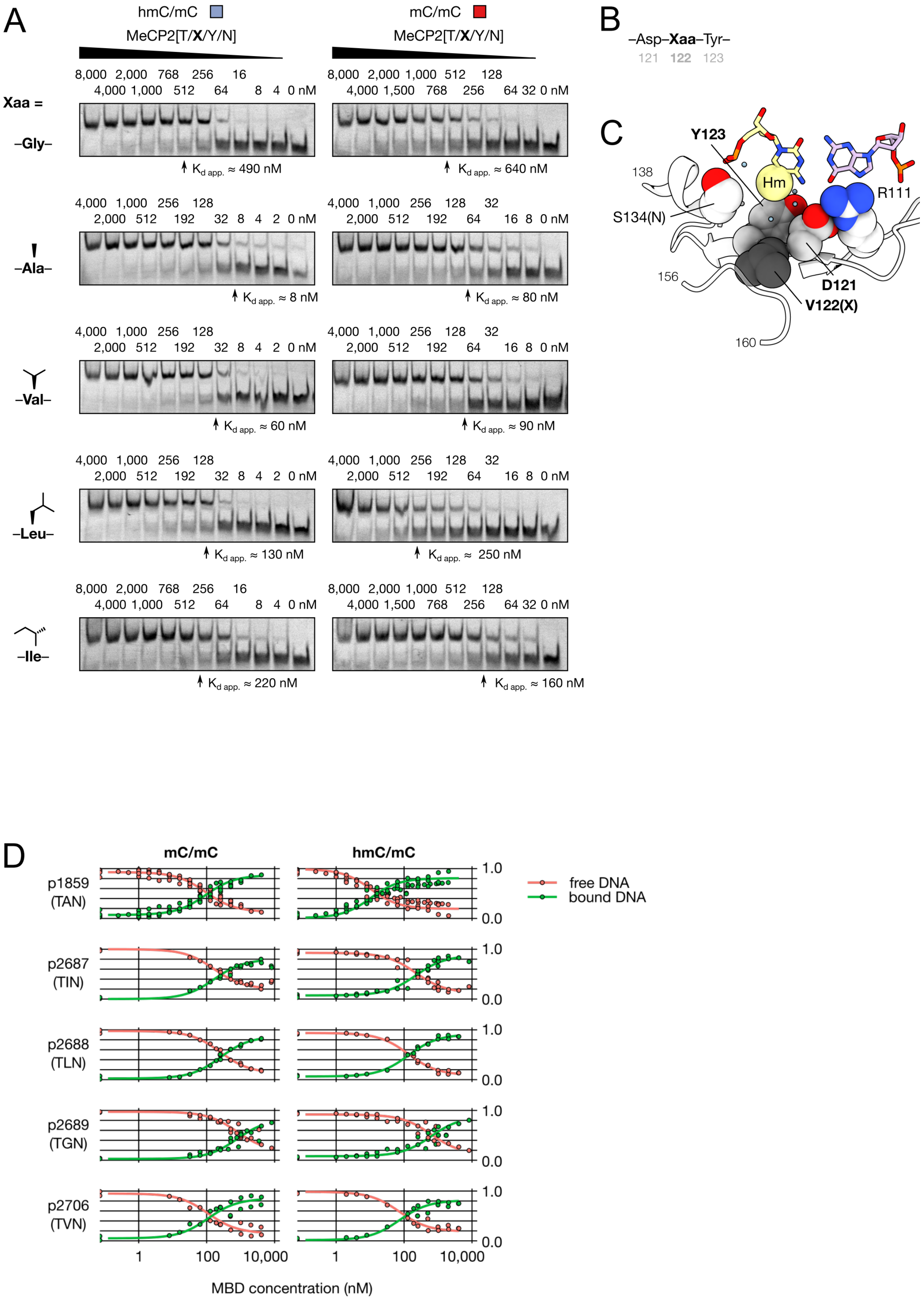
A
hmC/mC
mC/mC
MeCP2[T/X/Y/N]
Xaa =
–Gly–
–Ala–
–Val–
–Leu–
–Ile–
8,000 2,000 768 256 16
4,000 1,000 512 64 8 4 0 nM
8,000 2,000 1,000 512 128
4,000 1,500 768 256 64 32 0 nM
4,000 1,000 256 128
2,000 512 192 32 8 4 2 0 nM
4,000 1,000 256 128 32
2,000 512 192 64 16 8 0 nM
$K_{d\ app.}$ ≈ 490 nM
$K_{d\ app.}$ ≈ 640 nM
$K_{d\ app.}$ ≈ 8 nM
$K_{d\ app.}$ ≈ 80 nM
$K_{d\ app.}$ ≈ 60 nM
$K_{d\ app.}$ ≈ 90 nM
$K_{d\ app.}$ ≈ 130 nM
$K_{d\ app.}$ ≈ 250 nM
$K_{d\ app.}$ ≈ 220 nM
$K_{d\ app.}$ ≈ 160 nM
B
–Asp–Xaa–Tyr–
121 122 123
C
Y123
Hm
R111
138
S134(N)
156
D121
V122(X)
160
D
mC/mC
hmC/mC
p1859 (TAN)
p2687 (TIN)
p2688 (TLN)
p2689 (TGN)
p2706 (TVN)
1.0
0.0
1
100
10,000
MBD concentration (nM)
free DNA
bound DNA

Fig. S1 (previous page): Electrophoretic mobility shift assay (EMSA) for a series of MBD mutants. **(A)** EMSA gel images of a dilution series of MeCP2 (K109T/V122X/S134N) MBD with DNA duplexes. **(B)** Neighboring residues flanking the various mutations tested at position 122, **(C)** visualization of the V122X mutation site and neighboring amino acids in the structure of the complex (PDB 3c2i), and **(D)** Fitted curves to determine the apparent binding affinity (K_D) and associated model estimates shown in Supplementary Table S2. wt and TCN data are presented in identical form in reference (1).

Table S2: Model estimates from evaluating N = 3 EMSA gel shift images. Fitted data is shown in Figure S1D. wt and TCN data were presented in identical form in reference (1).

Protein	Probe	Term	Estimate (nM)	Standard error	Test statistic	p-value
**p1859**	mC/mC	K_D	9.211E+01	1.392E+01	6.615E+00	2.403E-08
**p1859**	mC/mC	B_{max}	8.000E-01	2.993E-02	2.673E+01	2.837E-31
**p1859**	mC/mC	b	6.980E-02	1.513E-02	4.612E+00	2.796E-05
**p1859**	hmC/mC	K_D	9.323E+00	1.853E+00	5.032E+00	5.937E-06
**p1859**	hmC/mC	B_{max}	8.000E-01	3.459E-02	2.313E+01	2.301E-29
**p1859**	hmC/mC	b	8.100E-03	3.016E-02	2.686E-01	7.893E-01
**p2526**	mC/mC	K_D	7.761E+01	1.140E+01	6.807E+00	1.205E-08
**p2526**	mC/mC	B_{max}	8.000E-01	2.860E-02	2.797E+01	3.383E-32
**p2526**	mC/mC	b	4.979E-02	1.549E-02	3.214E+00	2.296E-03
**p2526**	hmC/mC	K_D	2.670E+01	3.976E+00	6.716E+00	1.825E-08
**p2526**	hmC/mC	B_{max}	8.000E-01	2.518E-02	3.177E+01	2.351E-34
**p2526**	hmC/mC	b	6.017E-02	1.736E-02	3.465E+00	1.111E-03
**p2687**	mC/mC	K_D	1.669E+02	3.864E+01	4.319E+00	5.294E-04
**p2687**	mC/mC	B_{max}	8.000E-01	4.640E-02	1.724E+01	9.298E-12
**p2687**	mC/mC	b	0.000E+00	4.204E-02	0.000E+00	1.000E+00
**p2687**	hmC/mC	K_D	2.210E+02	3.997E+01	5.529E+00	8.371E-06
**p2687**	hmC/mC	B_{max}	8.030E-01	3.496E-02	2.297E+01	8.563E-19
**p2687**	hmC/mC	b	7.276E-02	1.875E-02	3.881E+00	6.364E-04
**p2688**	mC/mC	K_D	2.529E+02	2.428E+01	1.042E+01	2.907E-08
**p2688**	mC/mC	B_{max}	8.487E-01	2.048E-02	4.145E+01	6.887E-17
**p2688**	mC/mC	b	2.353E-02	1.415E-02	1.663E+00	1.171E-01

**p2688**	hmC/mC	K_D	1.335E+02	1.416E+01	9.426E+00	1.081E-07
**p2688**	hmC/mC	B_{max}	8.476E-01	2.051E-02	4.132E+01	7.206E-17
**p2688**	hmC/mC	b	6.318E-02	1.452E-02	4.351E+00	5.698E-04
**p2689**	mC/mC	K_D	7.866E+02	2.607E+02	3.018E+00	6.802E-03
**p2689**	mC/mC	B_{max}	8.000E-01	7.790E-02	1.027E+01	2.019E-09
**p2689**	mC/mC	b	2.743E-02	3.905E-02	7.025E-01	4.904E-01
**p2689**	hmC/mC	K_D	6.303E+02	1.886E+02	3.342E+00	2.616E-03
**p2689**	hmC/mC	B_{max}	8.000E-01	7.783E-02	1.028E+01	1.834E-10
**p2689**	hmC/mC	b	8.121E-02	2.551E-02	3.184E+00	3.863E-03
**p2706**	mC/mC	K_D	1.097E+02	4.828E+01	2.272E+00	3.822E-02
**p2706**	mC/mC	B_{max}	8.000E-01	8.249E-02	9.698E+00	7.468E-08
**p2706**	mC/mC	b	5.043E-02	6.886E-02	7.322E-01	4.753E-01
**p2706**	hmC/mC	K_D	7.209E+01	2.078E+01	3.469E+00	3.438E-03
**p2706**	hmC/mC	B_{max}	8.000E-01	4.455E-02	1.796E+01	1.493E-11
**p2706**	hmC/mC	b	1.509E-02	3.560E-02	4.238E-01	6.777E-01

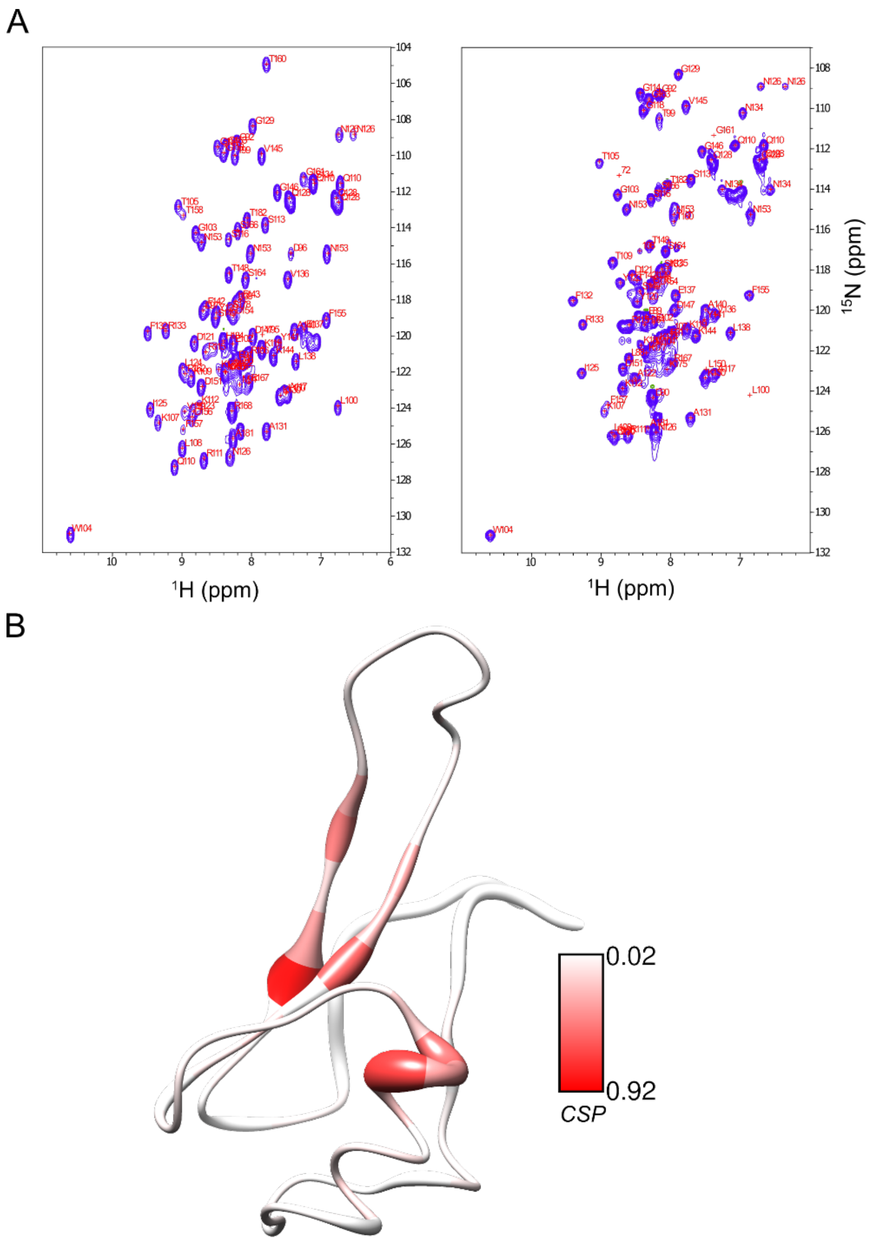

Fig. S2: (A) 2D [^{15}N–^{1}H]–HSQC of the wild-type (left) and TAN triple MBD mutant (right) at pH 6, 291K and 283 K, respectively, with assignments. The spectra were recorded with 128 and 2048 complex points along t_1 and t_2 dimensions. The assignments (deposited as BMRB entry 51020 for apo TAN) are indicated by the one-letter amino acid code followed by the corresponding sequence number along the primary protein sequence. **(B)** Chemical-shift perturbations between wild-type and triple MBD mutant (TAN) displayed on pdb 3c2i.

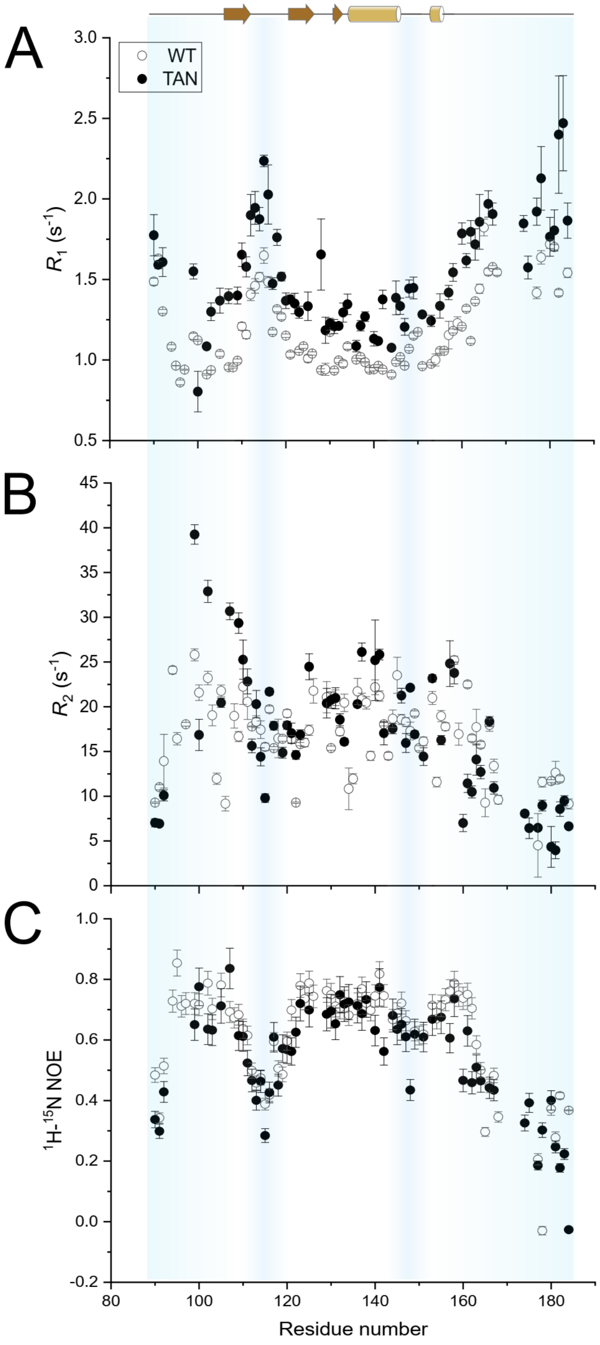

Fig. S3: Overlaid ^{15}N relaxation data of the wild-type (open circles) and triple mutant TAN (filled black circles) assessed as described in the Methods. (**A**) Longitudinal relaxation rates, R_1 ($1/T_1$), **(B)** transverse relaxation rates, R_2 ($1/T_2$), **(C)** [^{15}N, ^{1}H]-nOe, defined as I_{sat}/I_{eq}, where I_{sat} and I_{eq} are the intensities of peaks in the 2D experiments with and without proton saturation, respectively. The low values of R_2 and nOe for loop 1, consisting of nine residues (R111-K119) connecting β_1 and β_2, suggests its flexibility. Note that in A) the R_1 data of TAN were recorded at slightly higher temperature (293 K) but are overlaid here to better follow the trends (which are anyways highly consistent between the constructs).

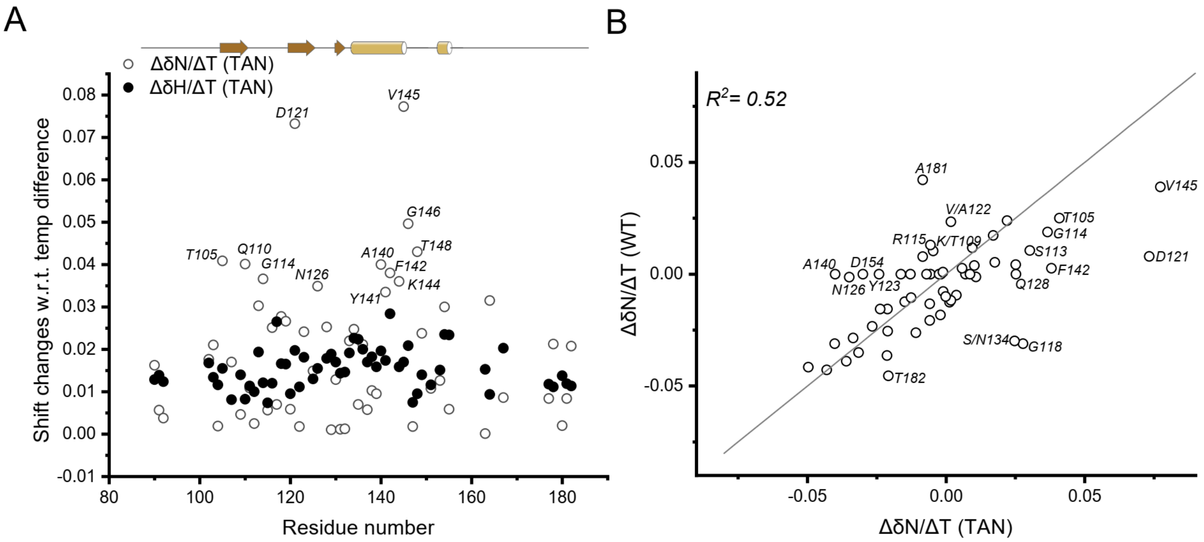

Fig. S4: Temperature-induced chemical-shift changes as a function of residue (ΔT (291-283 K)). **(A)** ΔδN/ΔT (open circles) and ΔδH/ΔT (filled black circles) as a function of sequence for the triple MBD mutant (TAN). **(B)** ΔδN/ΔT correlation plot of WT versus TAN.

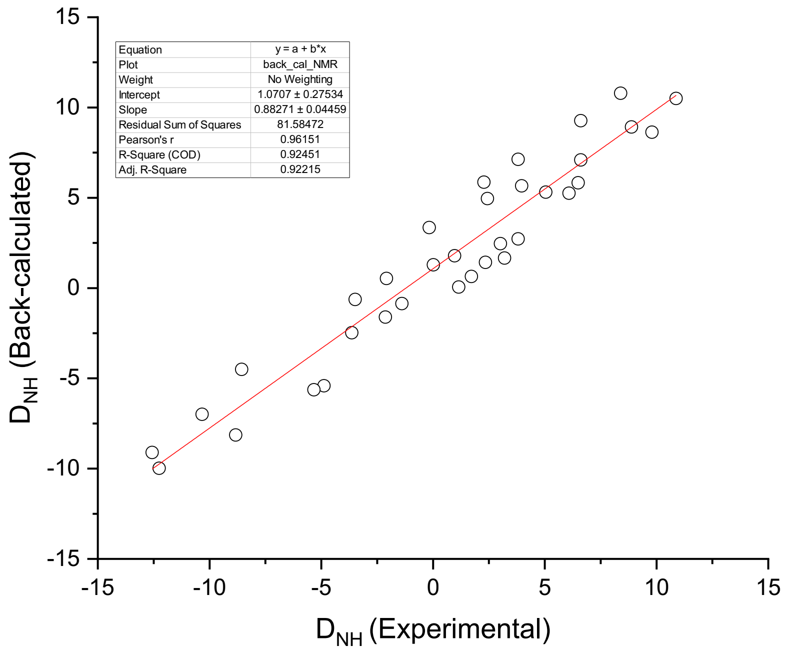

Fig. S5: Correlation between experimental and back-calculated backbone amide RDCs (D_{NH}), calculated from the ARIA-derived NMR structure of triple MBD mutant TAN, excluding residues 134, 140, 144, and 145 in helix α1. The experimental RDCs were recorded at 10°C.

Table S3: NMR structural statistics for the ensembles of 10 refined conformers of TAN, deposited to the PDB under accession code 8AJR, and TAN:hmC/mC, deposited as 8ALQ

**Conformationally restricting restraints**		
**Distance Restraints**	***TAN***	***TAN:hmC/mC***
Total	698	375
Intraresidue ([i = j])	180	102
Sequential ([i – j] = 1)	179	125
medium-range (1 < [i – j] < 5)	148	62
long-range ([i – j] ≥ 5)	191	86
Dihedral angle restrains (ϕ and φ)	108	114
Disulfide restraints	0	0
No. of restraints per residue	10	6.7
**Model quality**		
RMSD backbone atoms (Å)	1.0	1.0
RMSD heavy atoms (Å)	1.4	1.4
**MolProbity Ramachandran statistics**		
Most favored region (%)	94.9	93.8
Allowed region (%)	5.1	6.2
Additionally allowed region (%)	0.0	0.0
Disallowed region (%)	0.0	0.0
**Global quality scores (raw/Z score)**		
Verify3D	0.27 / -3.05	0.17 / -4.65
PROCHECK (ϕ-ψ)	-0.30 / -0.87	-0.40 / -1.26
PROCHECK (all)	-0.31/ -1.83	-0.43 / -2.54
MolProbity clash score	12.3 / -0.59	11.51 / -0.45
**Model contents**		
Total no. of residues	104	104
BMRB accession number	51020	34745
PDB ID code	8AJR	8ALQ

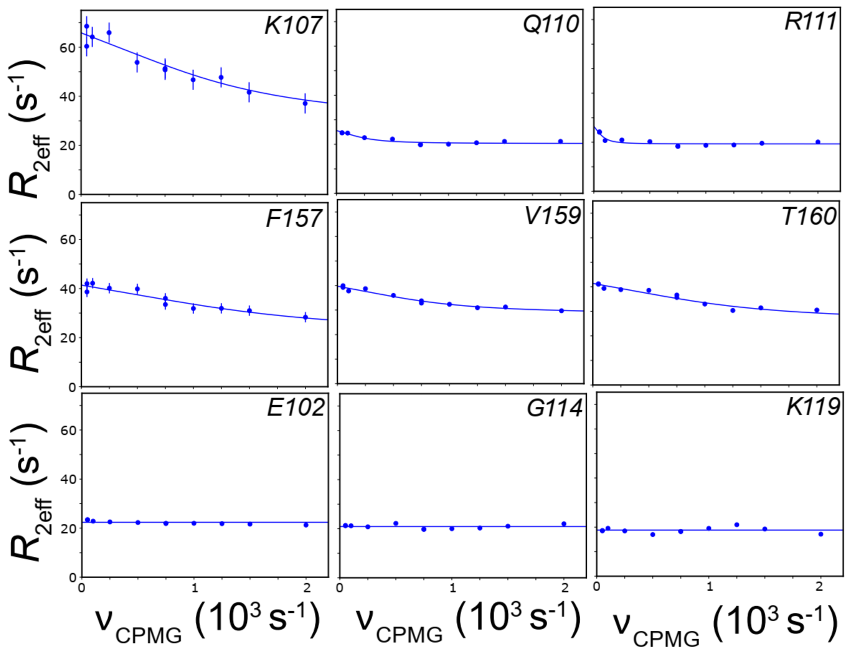

Fig. S6: Selected ^{15}N relaxation dispersion profiles of the wt MBD: $R_{2\text{eff}}$ versus CPMG frequency from individual fittings.

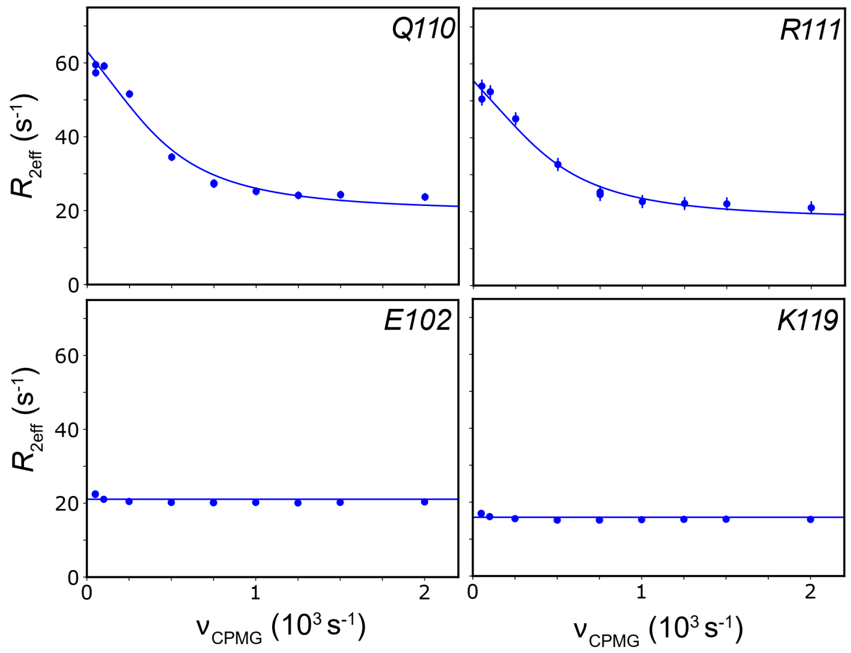

Fig. S7: Selected ^{15}N relaxation dispersion profiles of the triple MBD mutant (TAN).

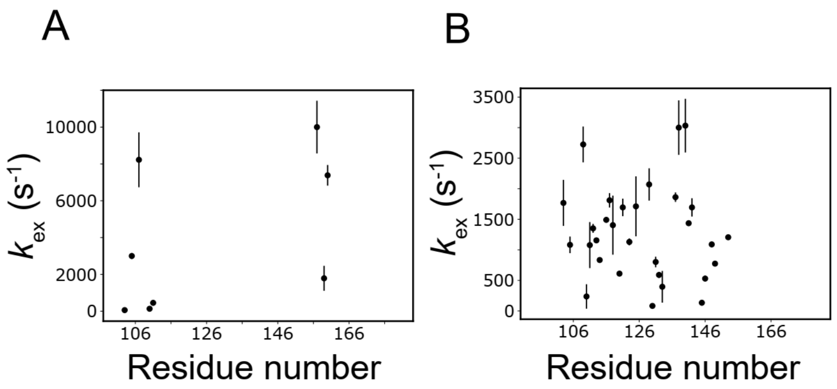

Fig. S8: k_{ex} as a function of residue for the **(A)** wt and **(B)** triple MBD mutant (TAN).

Table S4: Model selection, kinetic and thermodynamic exchange parameters from ^{15}N CPMG recorded on apo-TAN at two fields, 800 and 700 MHz, at 283 K.

**Res. #**	**Model**	**R_2 (s^{-1})**	**Err.**	**k_{ex} (s^{-1})**	**Err.**	**R_{ex} exp. (rad/s)**	**Err.**	**p_E**	**Err.**	**δω [ppm]**	**Err.**
88	1	16.62	2.35								
89	1	15.00	0.02								
90	1	15.00	0.04								
92	1	15.00	0.21								
102	1	23.44	0.07								
103	3	24.28	1.59	1132	229.4	10.17	0.19	0.014	0.001	3.12	0.10
105	3	27.02	1.28	1315	147.1	8.03	0.29	0.372	0.241	0.42	0.48
106	3	23.70	2.11	2063	266.8	10.13	0.29	0.100	0.040	0.97	0.65
108	3	23.46	3.38	1640	88.63	11.46	0.19	0.091	0.032	0.98	0.34
110	2	23.58	1.66	2168	86.16	10.69	0.16				
111	3	23.77	1.09	2258.	227.3	10.44	0.38	0.100	0.038	1.03	0.55
112	1	23.43	1.03								
113	3	16.34	1.50	1053	101.6	7.49	0.08	0.010	0.0003	3.07	0.08
114	3	18.50	0.61	889.2	42.94	8.04	0.14	0.100	0.028	0.58	0.16
115	1	17.26	2.05								
117	3	18.37	0.85	1078	60.61	7.67	0.11	0.015	0.0010	2.08	0.10
118	3	20.54	0.34	902.7	26.50	9.27	0.12	0.100	0.018	0.64	0.10
119	1	20.69	1.82								
121	2	26.62	2.65	3681	157.5	10.75	0.27				

122	1	21.64	1.36								
123	3	23.36	2.01	1283	251.4	5.26	0.28	0.0079	0.0011	2.64	0.22
125	3	26.65	1.50	1415	255.9	8.79	0.33	0.012	0.0021	2.92	0.27
129	1	26.72	0.32								
130	3	22.88	1.02	1584	50.55	4.99	0.02	0.023	0.0067	1.24	0.15
131	3	24.47	0.11	1001	113.1	5.89	0.21	0.033	0.031	0.93	0.28
132	3	26.02	0.66	51.09	24.46	10.11	2.91	0.301	0.092	0.40	0.07
133	3	25.70	2.25	187.14	130.4	4.26	0.30	0.025	0.027	1.42	0.10
134	3	27.27	1.45	2297	166.2	10.17	0.20	0.076	0.038	1.17	0.60
136	1	26.64	0.75								
137	2	26.85	0.38	1044	37.78	7.49	0.10				
138	3	27.72	1.14	507.6	96.90	6.58	0.33	0.016	0.002	2.14	0.14
140	2	25.14	2.11	3930	1390	7.10	0.17				
141	3	26.20	0.09	1252	270.4	3.94	0.15	0.004	0.001	5.78	0.23
142	3	25.92	2.08	572.2	95.47	6.15	0.41	0.080	0.011	0.46	0.15
144	1	25.69	0.14								
145	2	26.96	1.55	6306	115.8	14.45	0.14				
147	1	21.13	0.79								
148	3	19.54	0.57	1090	52.59	9.33	0.10	0.100	0.024	0.69	0.17
149	2	22.37	1.57	500.0	10.94	7.40	0.15				
150	1	24.56	0.49								
151	1	22.77	0.90								
153	3	24.41	1.94	891.1	96.89	6.84	0.18	0.100	1.2E-8	0.53	0.02
155	1	24.06	0.75								
160	3	10.24	0.26	38.71	0.85	3.48	0.08	0.100	9.5E-9		
161	3	15.00	0.03	281. 7	14.17	9.47	0.24	0.049	0.0013	3.87	0.23
163	1	18.02	0.65							0.89	0.02
164	1	17.65	0.53								
166	2	24.16	0.44	844.4	52.99	12.72	0.30				
167	1	15.98	0.74								
174	1	15.00	0.07								
175	1	15.60	0.31								
178	1	15.09	0.04								
180	1	15.00	0.10								
181	1	15.37	0.19								
182	1	15.00	0.08								
183	1	15.00	0.24								

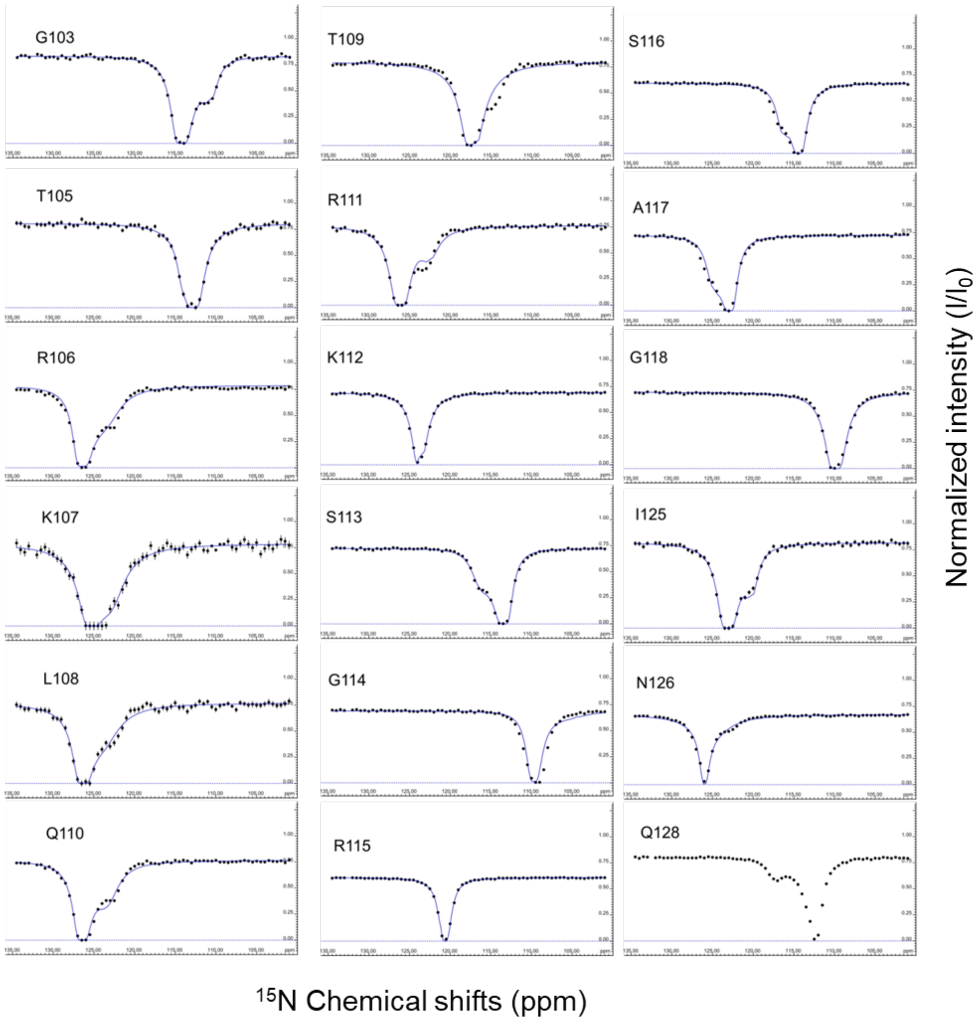

Fig S9: Representative ^{15}N CEST profiles of the indicated amide groups of TAN MBD mutant. The major dip corresponds to the ground state, whereas the minor dips correspond to the energetically excited state. The ratio I/I_0 is plotted, where I is the intensity after irradiation and I_0 is the intensity without irradiation. The ^{15}N CEST experiments were recorded at 800 MHz using a B_1 saturating field of 30 Hz at 283K.

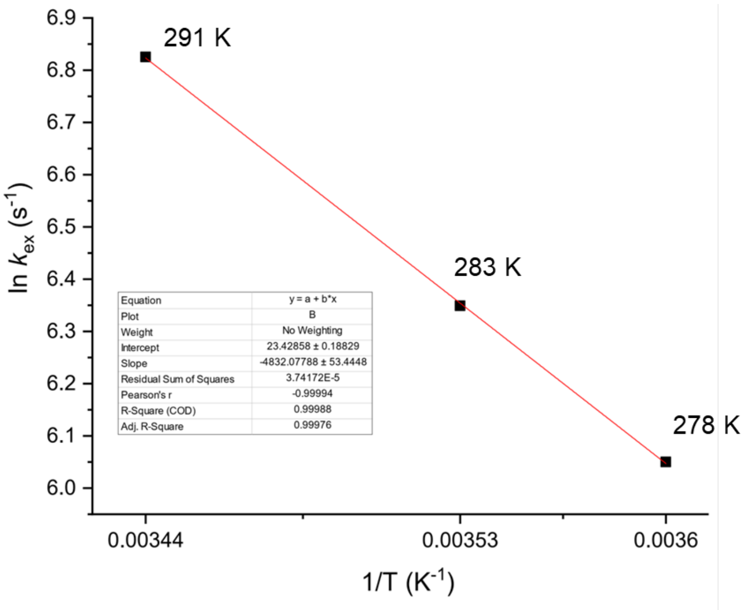

Fig. S10: Arrhenius plot of the k_{ex} measured by the CEST experiments in (Fig. S9) for the three temperatures (278, 283, and 291K) indicated to determine the activation energy of the ground state transition to the excited-state conformation. The ^{15}N CEST experiments were recorded at 800 MHz using a B_1 saturating field of 30 Hz.

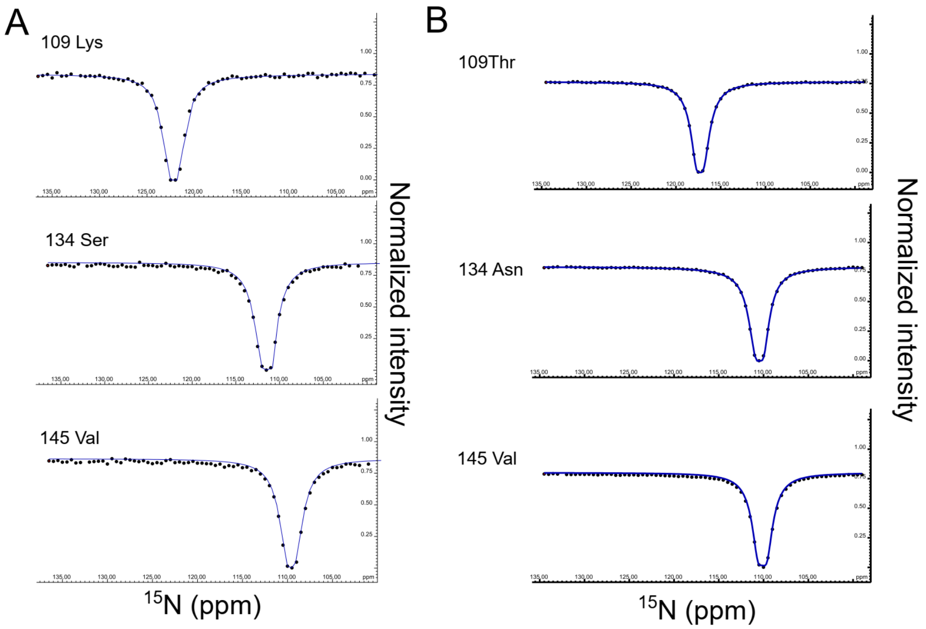

Fig. S11: Representative ^{15}N CEST profiles of the indicated amide groups of **(A)** wt and **(B)** double-mutant (TVN) MBD of MeCP2. Normalized ratio, the ratio I/I_0 is plotted, where I is the intensity after irradiation and I_0 is the intensity without irradiation. The ^{15}N CEST experiments were recorded at 800 MHz using a B_1 saturating field of 30 Hz at 291 K.

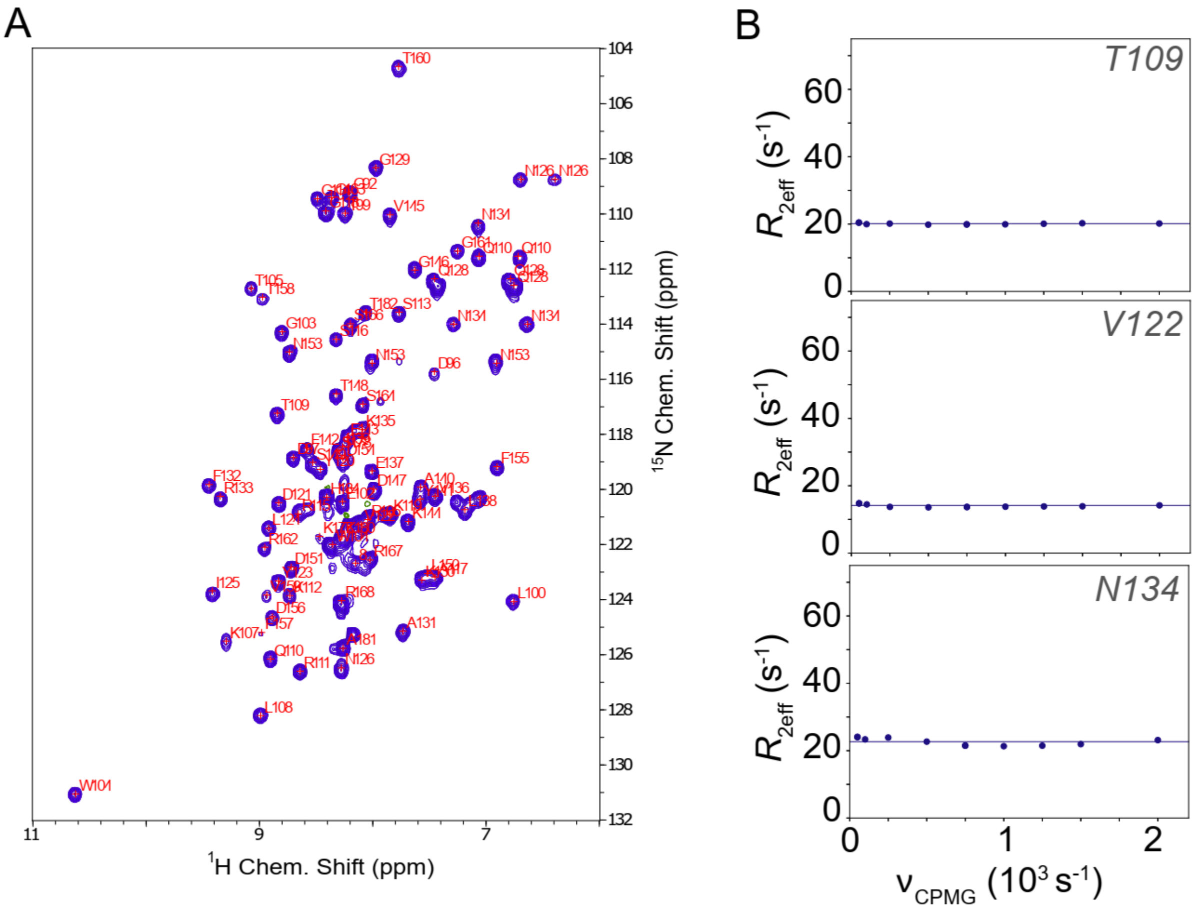

Fig. S12: Structure and dynamics characterization of the double mutant MBD (TVN). **(A)** Assigned 2D ^{15}N-^{1}H HSQC of double mutant TVN. **(B)** Selected ^{15}N CPMG relaxation dispersion profiles of the double mutant TVN: R_{2eff} versus CPMG frequency.

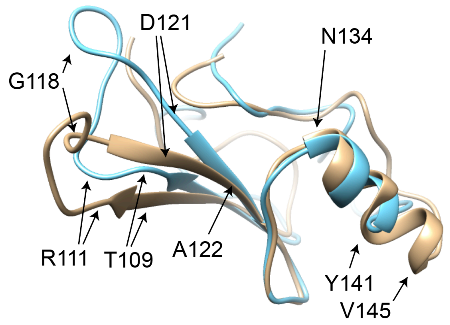

Fig. S13: Incident of partial unfolding in MD simulations as seen in one out of five trajectories. Marked are mutated residues and residues of interest for structural comparison. The starting structure is shown in brown, a frame at around 2 μs is shown in blue.

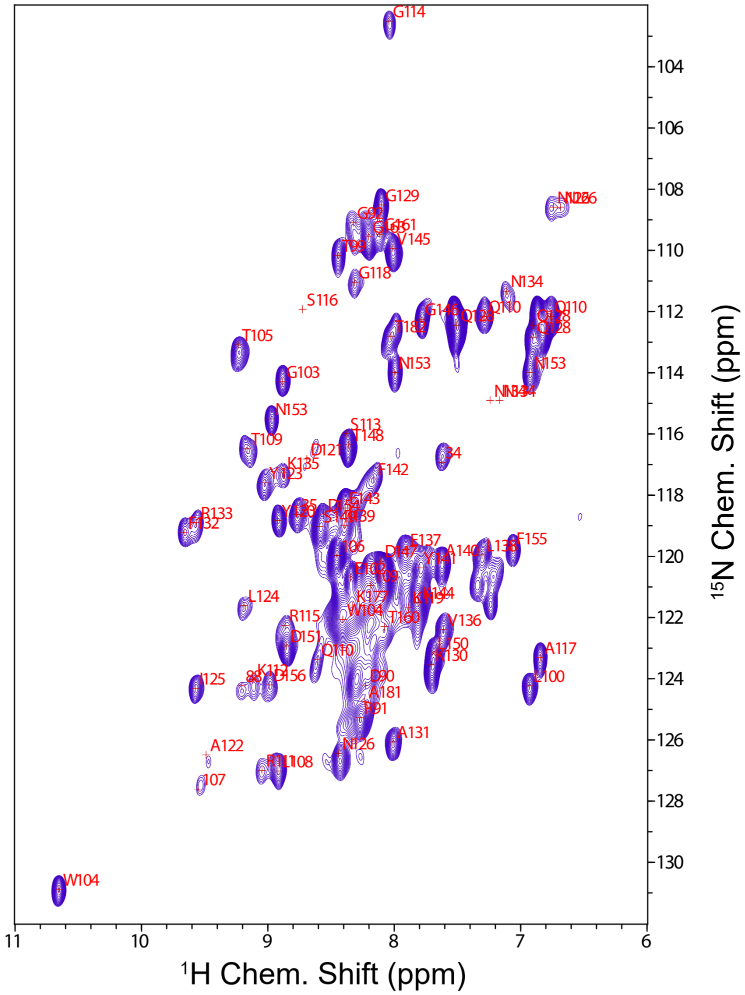

Fig. S14: Assigned 2D ^{15}N-^{1}H HSQC of hmC/mC DNA-bound TAN mutant. The assignments have been deposited into the BMRB under accession code 34745.

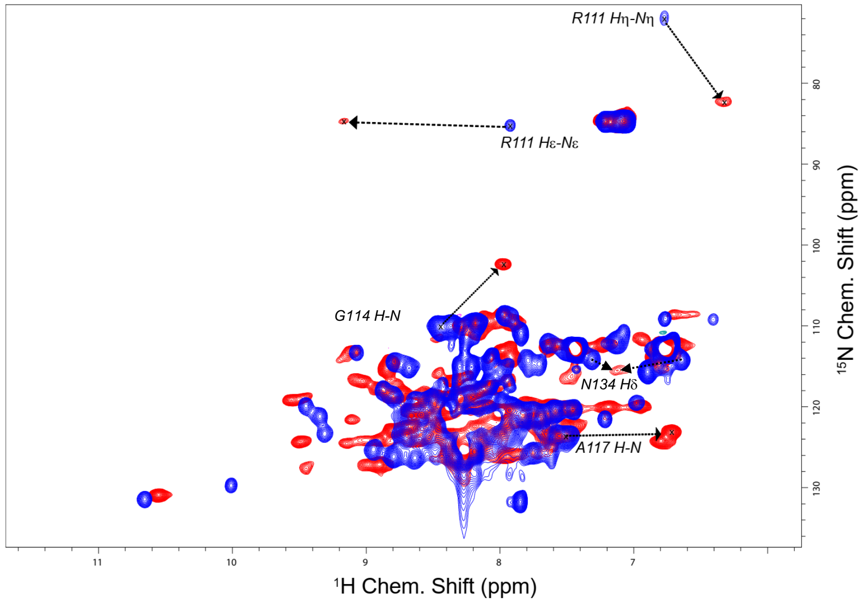

Fig. S15: Selected chemical-shift changes in apo TAN (red) versus TAN:hmC/mC DNA complex (blue). The ^{1}Hϵ of R111 shows its ^{1}H resonance strongly downfield-shifted from 8 ppm to 9.3 ppm. Similarly, G114 ^{15}N is moved upfield from 109.2 ppm to 102.6 ppm, A117 $^{1}H^{N}$ from 7.36 to 6.84 ppm, R111 ^{15}Nη is shifted downfield by 11 ppm, and N134 Hδ is shifted by around 0.3 ppm. The N134 Hδ resonance of the complex represents either $H\delta_{21}$ or $H\delta_{22}$, which remains ambiguous as upon complex formation one of the two resonances is exchange-broadened.

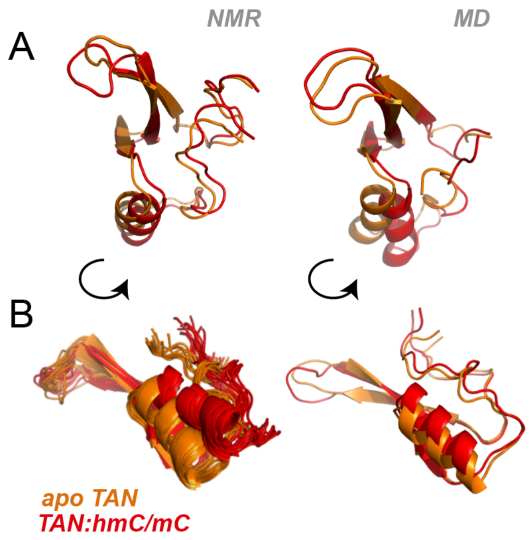

Fig. S16: Overlays of apo TAN and complex seen by either NMR (left) or MD (right). **A)** Visualization of the displacement of the N-terminal side of helix α1 (similar between NMR and MD). **B)** Visualization of the angle of the helix α1, deviating in the NMR structure calculation by 5-15 °. As measurement of residual dipolar couplings was unsuccessful in the presence of DNA, the experimental complex structure from NMR is not fully reliable with respect to the relative orientation of the secondary-structural elements. All structures are overlaid with respect to the β1 sheet.

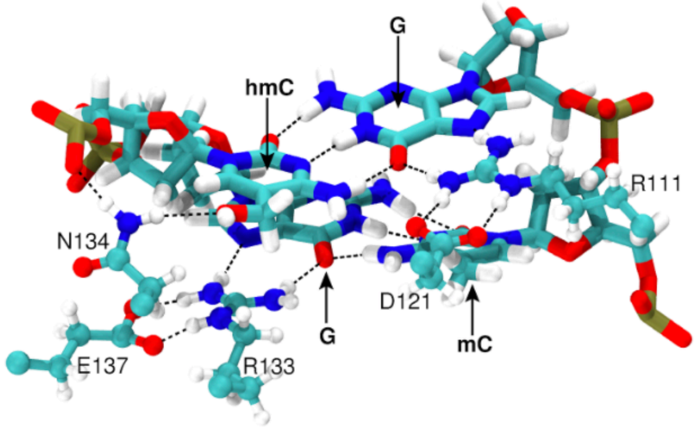

Fig. S17: Visualization of the specific interactions of the hmC/mC reader TAN with its target DNA, including the salt bridges that are temporarily formed between E137 and R133 and between D121 and R111, as seen in MD simulations.

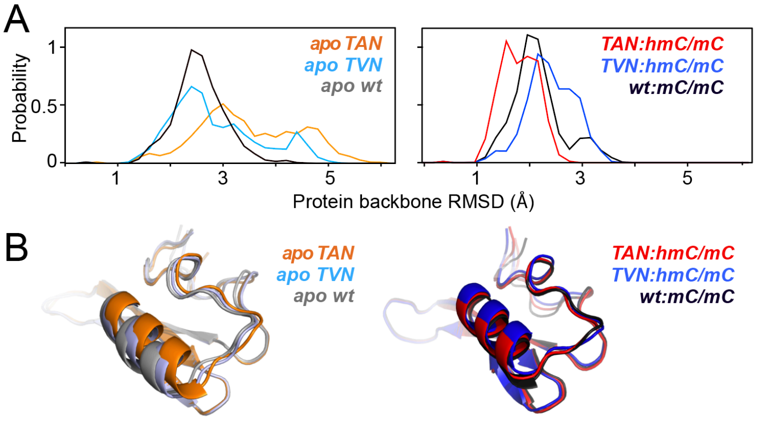

Fig. S18: Differential backbone plasticity of wt, TVN, and TAN mutants in their apo form and in complex with (respectively matching) dsDNA, as seen in MD simulations. **A)** Relative occurrence of deviations (overall backbone RMSD) from the average structure. **B)** Additional combinations of overlaid average structures from 2.5 µs MD simulations of wt, TAN, and TVN readers in apo form or in complex with (matching) target DNAs, adding to main text Fig. 6C.

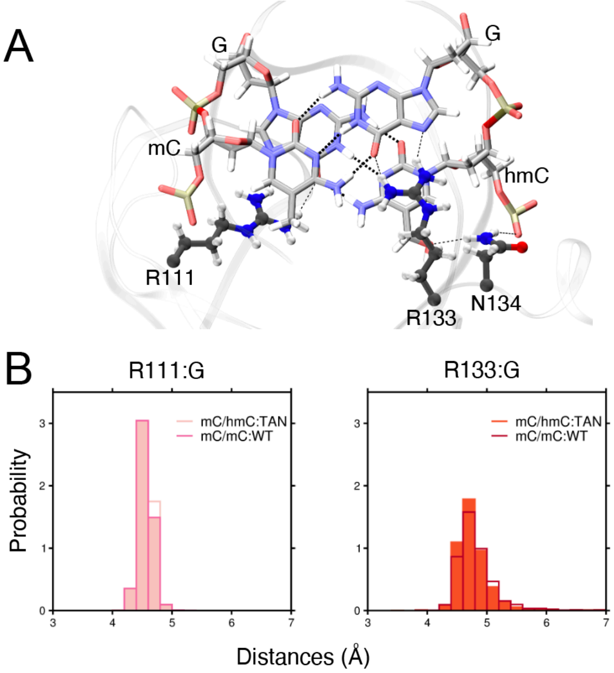

Fig. S19: H-bond stability between Arg and guanosine in the complex formed between TAN and hmC/mC DNA, determined in the MD simulations both for R111 (left) and R133 (right).

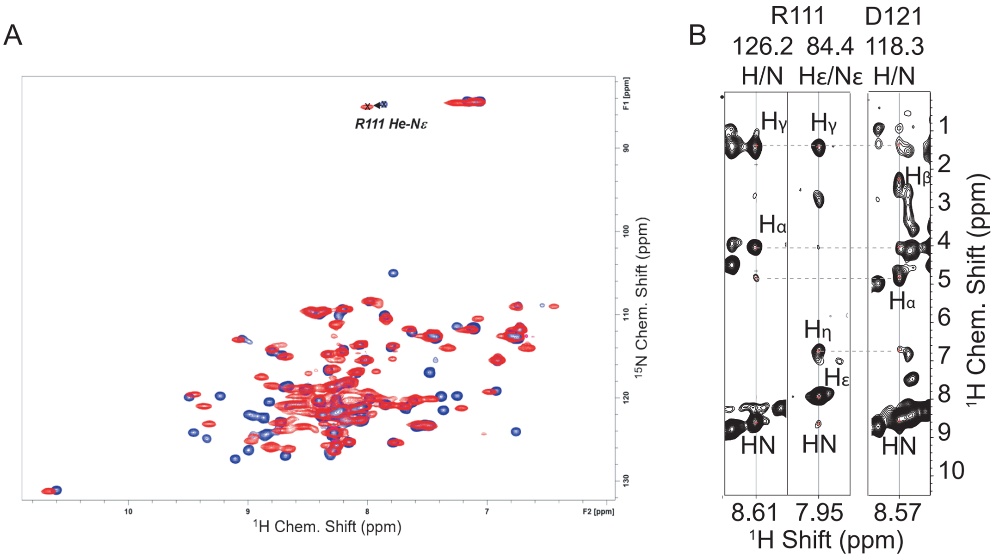

Fig. S20: Identification of the R111-D121 salt-bridge in the wt and TAN mutant MBD. **A)** Overlaid ^{15}N-^{1}H HSQC of wt (in blue) and triple MBD mutant (TAN, in red) showing mutation-specific changes to the Hε-Nε Arg111 sidechain shift, a key residue whose mutation causes Rett syndrome. **B)** R111 sidechain contacts to D121 observed in ^{15}N-edited NOESY-HSQC spectra.

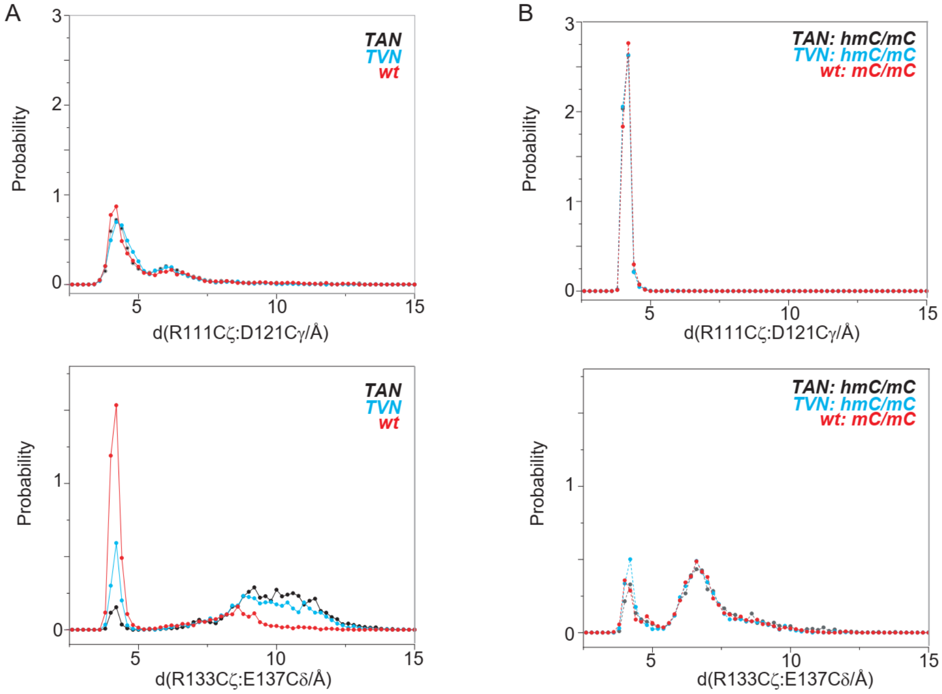

Fig. S21: Salt bridges formed in the TAN, TVN, and wt MBD without (A) and with DNA (B). (Same as in Main Text Fig. 6F, but overlaying either all apo proteins or complexes, respectively, and including the TVN mutant.)

References:

1. B. C. Buchmuller *et al.*, Evolved DNA Duplex Readers for Strand-Asymmetrically Modified 5-Hydroxymethylcytosine/5-Methylcytosine CpG Dyads. *J. Am. Chem. Soc.* **144**, 2987-2993 (2022).
2. J. Marley, M. Lu, C. Bracken, A method for efficient isotopic labeling of recombinant proteins. *J Biomol. NMR* **20**, 71-75 (2001).
3. R. A. Venters, B. T. Farmer, C. A. Fierke, D. S. Leonard Characterizing the Use of Perdeuteration in NMR Studies of Large Proteins: 13C, 15N and 1H Assignments of Human Carbonic Anhydrase II. *J. Mol. Biol.* **264**, 1101–1116 (1996).
4. R. I. Wakefield *et al.*, The solution structure of the domain from MeCP2 that binds to methylated DNA. *J. Mol. Biol.* **291**, 1055-1065 (1999).
5. W. Rieping *et al.*, ARIA2: automated NOE assignment and data integration in NMR structure calculation. *Bioinformatics* **23**, 381-382 (2007).

6. M. Ottiger, F. Delaglio, A. Bax, Measurement of J and Dipolar Couplings from Simplified Two-Dimensional NMR Spectra. *J. Magn. Reson.* **131**, 373-378 (1998).
7. M. Zweckstetter, NMR: prediction of molecular alignment from structure using the PALES software. *Nat. Protoc.* **3**, 679-690 (2008).
8. S. Meiboom, D. Gill, Modified Spin-Echo Method for Measuring Nuclear Relaxation Times. *Rev. Sci. Inst.* **29**, 688-691 (1958).
9. R. Keller (2004) Optimizing the process of nuclear magnetic resonance spectrum analysis and computer aided resonance assignment. (PhD thesis, ETH Zurich).
10. W. F. Vranken *et al.*, The CCPN data model for NMR spectroscopy: development of a software pipeline. *Proteins* **59**, 687–696 (2005).
11. F. A. A. Mulder, N. R. Skrynnikov, B. Hon, F. W. Dahlquist, L. E. Kay, Measurement of Slow (μs−ms) Time Scale Dynamics in Protein Side Chains by 15N Relaxation Dispersion NMR Spectroscopy: Application to Asn and Gln Residues in a Cavity Mutant of T4 Lysozyme. *J. Am. Chem. Soc.* **123**, 967-975 (2001).
12. W. Lee, M. Tonelli, J. L. Markley, NMRFAM-SPARKY: enhanced software for biomolecular NMR spectroscopy. *Bioinformatics* **31**, 1325-1327 (2015).
13. M. Bieri, P. R. Gooley, Automated NMR relaxation dispersion data analysis using NESSY. *BMC Bioinformatics* **12**, 421 (2011).
14. A. Mazur, B. Hammesfahr, C. Griesinger, D. Lee, M. Kollmar, ShereKhan—calculating exchange parameters in relaxation dispersion data from CPMG experiments. *Bioinformatics* **29**, 1819-1820 (2013).
15. P. Vallurupalli, G. Bouvignies, L. E. Kay, Studying "invisible" excited protein states in slow exchange with a major state conformation. *J. Am. Chem. Soc.* **134**, 8148–8161 (2012).
16. H. M. McConnell, Reaction Rates by Nuclear Magnetic Resonance. *J. Chem. Phys.* **28**, 430-431 (1958).
17. Y. Shen, F. Delaglio, G. Cornilescu, A. Bax, TALOS+: a hybrid method for predicting protein backbone torsion angles from NMR chemical shifts. *J. Biomol. NMR* **44**, 213-223 (2009).
18. L. Schrödinger, The PyMOL Molecular Graphics System, Version-1.8. (2015).
19. K. L. Ho *et al.*, MeCP2 Binding to DNA Depends upon Hydration at Methyl-CpG. *Mol. Cell* **29**, 525-531 (2008).
20. E. F. Pettersen *et al.*, UCSF Chimera—A visualization system for exploratory research and analysis. *J. Comput. Chem.* **25**, 1605-1612 (2004).
21. A. Pérez *et al.*, Refinement of the AMBER force field for nucleic acids: improving the description of alpha/gamma conformers. *Biophys. J.* **92**, 3817-3829 (2007).
22. M. Zgarbová *et al.*, Refinement of the Cornell et al. Nucleic Acids Force Field Based on Reference Quantum Chemical Calculations of Glycosidic Torsion Profiles. *J. Chem. Theory Comput.* **7**, 2886-2902 (2011).
23. M. J. Abraham *et al.*, GROMACS: High performance molecular simulations through multi-level parallelism from laptops to supercomputers. *SoftwareX* **1-2**, 19-25 (2015).
24. H. J. C. Berendsen, J. P. M. Postma, A. DiNola, J. R. Haak, Molecular dynamics with coupling to an external bath. *J. Chem. Phys.* **81**, 3684–3690 (1984).
25. S. Páll, B. Hess, A flexible algorithm for calculating pair interactions on SIMD architectures. *Comput. Phys. Commun.* **184**, 2641-2650 (2013).
26. T. Darden, D. York, L. Pedersen, Particle mesh Ewald: An N·log(N) method for Ewald sums in large systems. *J. Chem. Phys.* **98**, 10089-10092 (1993).
27. B. Hess, P-LINCS: A parallel linear constraint solver for molecular simulation. *J. Chem. Theory Comp.* **4**, 116–122 (2007).
28. S. Miyamoto, P. A. Kollman, SETTLE: An analytical version of the SHAKE and RATTLE algorithms for rigid water models. *J. Comp. Chem.* **13**, 952–962 (1992).
